# AI-Augmented Multi-Omics for Abiotic Stress Responses: A New Frontier in Plant Hormone Systems Biology

**DOI:** 10.3390/plants15142185

**Published:** 2026-07-16

**Authors:** Yao Zhang, Xinyi Cao, Liming Yang, Jinhui Chen, Delight Hwarari

**Affiliations:** 1 State Key Laboratory of Tree Genetics and Breeding, College of Life Sciences, Nanjing Forestry University, Nanjing 210037, China; 202312082@njfu.edu.cn (Y.Z.); 2411801201@njfu.edu.cn (X.C.); yangliming@njfu.edu.cn (L.Y.); 2 State Key Laboratory of Tree Genetics and Breeding, College of Forestry, Nanjing Forestry University, Nanjing 213007, China

**Keywords:** plant hormones, multi-omics integration, forest trees, stress physiology, abiotic stress resilience, machine learning, deep learning

## Abstract

Plant phytohormone networks control growth, dormancy, and stress responses throughout plants’ long lifespans and heterogeneous tissues. While multi-omics approaches have advanced the understanding of these regulatory systems, their application remains constrained by low spatial resolution, static sampling strategies, and limited capacity to capture nonlinear, context-dependent interactions. These limitations are especially evident in forest tree species, where hormone gradients change dynamically across developmental stages, seasons, and environmental conditions. The emergence of Artificial Intelligence (AI), including its subsets deep learning (DL) and machine learning (ML), provides valuable solutions to overcome these barriers. These solutions involve integrating high-dimensional data, reconstructing spatiotemporal hormone architectures, and developing predictive network models that regulate stress resilience and growth. This review highlights recent research on integrative omics in forest hormone biology and discusses how AI technologies overcome the barriers in traditional multi-omics approaches. Additionally, it outlines future directions for developing translational tools to support sustainable forestry production and management.

## 1. Introduction

Plants, including forest trees, rely on complex hormonal systems for long-term growth, development, and stress response. Phytohormones such as auxins, cytokinins, gibberellins, ABA, ethylene, jasmonates, salicylic acid, and brassinosteroids regulate plant stress responses [1]. These hormones use intricate signaling networks to link environmental stressors and maintain tree homeostasis under extreme conditions. Unlike annual crops, trees continually reprogram hormonal pathways across seasons, stages, and tissues, making their regulatory systems dynamic and complex [1]. Over the past decade, multi-omics techniques have improved our understanding of hormone-driven processes in forest biology. Research across genomics, transcriptomics, proteomics, phosphoproteomics, metabolomics, and epigenomics has identified regulators of hormone production, transport, signaling, post-translational regulation, metabolic changes, and stable epigenetic configurations influencing hormone responsiveness [2,3].

Despite these advances, traditional multi-omics integration remains insufficient to decode abiotic stress responses in perennial trees. Since bulk datasets obscure tissue-specific ABA dynamics, static sampling fails to capture rapid drought-induced signaling waves, and correlation-based models cannot resolve the nonlinear hormone crosstalk that underpins adaptation to drought, heat, cold, and salinity [4]. Furthermore, the complexity and variability of multi-omics datasets, coupled with incomplete annotations, pose challenges for standard statistical integration in non-model forest species [5]. These issues hinder the development of mechanistic, predictive models of hormone signaling, especially under complex environmental conditions.

Therefore, Artificial Intelligence (AI), including machine learning (ML), deep learning (DL), graph neural networks (GNNs), and protein language models (PLLMs), has significant potential to address these challenges. AI-augmented multi-omics now integrate high-dimensional data to predict hormone-regulated abiotic stress responses, reconstruct signaling networks from incomplete omics layers, and infer gene function in non-model forest species with sparse annotations [6]. Recent applications demonstrate that AI architectures, particularly graph neural networks (GNNs), variational autoencoders (VAEs), and transfer learning frameworks, enable systems-level integration of plant stress responses that traditional statistical approaches cannot match [7]. GNN-based models have reconstructed non-linear hormone crosstalk networks, such as ABA–auxin interactions under drought, by learning from multilayered omics graphs, thereby capturing regulatory logic that correlation-based methods overlook [8]. VAE frameworks have successfully integrated fragmented cold-stress datasets across transcriptomic, metabolomic, and epigenomic modalities, inferring latent biological states from incomplete observations. Transfer learning has enabled cross-species prediction of stress regulators in non-model conifers by leveraging pre-trained models from well-annotated angiosperms, thereby overcoming the data scarcity that has traditionally limited crop research [7,8].

These advances illustrate AI’s capacity for hypothesis-free pattern discovery, cross-modal data fusion, and knowledge transfer across phylogenetic boundaries, capabilities that extend far beyond the linear modeling and pathway enrichment paradigms of traditional multi-omics analysis.

This review discusses recent multi-omics research on forest species and shows how AI-enhanced methods advance understanding of hormone biology beyond traditional approaches. It highlights emerging computational innovations and critical gaps that must be addressed to create predictive, mechanistic, and climate-resilient hormone models. By merging multi-omics and AI, the review explores opportunities for next-generation tools for sustainable forestry, precision breeding, and climate resilience.

## 2. Multi-Omics Foundations and Insights into Hormonal Regulation in Forest Systems

A comprehensive analysis of hormone-mediated processes in forest trees requires examining multiple biological layers. Omics approaches, such as genomics, transcriptomics, metabolomics, and epigenomics, provide partial insights into the mechanisms underlying tree stress adaptation. Although individual omics studies yield significant findings, integrating omics data enables a system-level analysis of hormonal signaling in forest trees [9].

### 2.1. Genomics and Transcriptomics: Mapping Stress Hormone-Responsive Gene Networks

**Figure 1:**
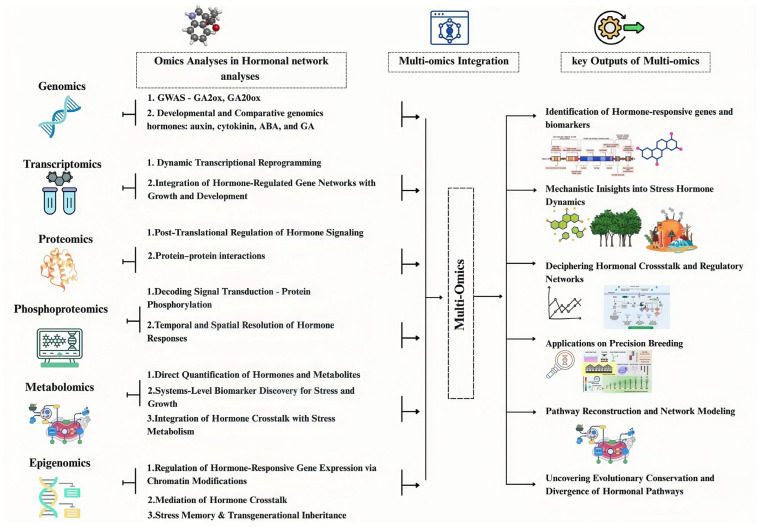
Integrative multi-omics framework for analyzing hormonal networks and making biological inferences. This diagram illustrates how genomics, transcriptomics, proteomics, phosphoproteomics, metabolomics, and epigenomics contribute to the understanding of plant hormonal signaling. Each omics layer uncovers different regulatory processes, from cis-element structures and transcriptional changes to post-translational modifications and hormone measurements, enabling a systems-level view of hormone-driven growth and stress responses. Centralized multi-omics integration helps identify hormone-responsive genes and biomarkers, reconstruct signaling pathways, and model regulatory networks. These insights provide a mechanistic understanding of hormonal crosstalk, evolutionary conservation, and targeted breeding strategies. This figure was drawn using Canva online software (https://www.canva.com/).

Genomics identifies key hormonal regulators of plant growth and stress adaptation (Figure 1). It uses techniques like whole-genome sequencing (WGS) to generate foundational genome assemblies for large, repetitive, and heterozygous genomes [10,11]. Examples include short- and long-read sequencing, chromosome-scale scaffolding, and haplotyping and phasing tools [11]. Approaches in genome annotation and gene family characterization include structural and functional annotation, gene family expansion analyses, and discovery of promoters and cis-regulatory motifs.

Additionally, techniques in population and evolutionary genomics help reveal hormone pathways conserved across different lineages, which are vital for tree breeding and improvement [12]. These include SNP genotyping (GBS, RAD-seq), genome–environment association (GEA), and scanning for selective sweeps and local adaptation. Other techniques, such as structural genomics (SV detection, pangenome construction) and functional genomics (CRISPR/Cas, RNA interference, VIGS), enable direct analysis of gene expression in hormone pathways, as well as comparative, phylogenomic, and integrative approaches (eQTL, GWAS) [13].

Transcriptomics quantifies genome-wide mRNA abundance across spatial, temporal, and biological axes to map hormone-responsive gene networks under abiotic stress (Figure 1). Bulk RNA-seq of dissected organs such as roots, leaves, and meristems establishes tissue-specific expression baselines and captures stress-related changes. Time-series profiling (minutes to weeks) resolves regulatory waves, with early transcription factors (DREB, NAC, WRKY, bZIP) activating downstream genes [14]. Comparative transcriptomics across stresses (drought, salinity, cold, heat, combinations) identifies stress-specific adaptations and hormone pathway components with universal versus specialized functions. Long-read transcriptomics (Iso-Seq, Oxford Nanopore) produces full-length transcripts, resolves isoforms, and improves gene annotation, showing that abiotic stress induces extensive isoform switching in hormone signaling components, including ABA receptors, stress-responsive transcription factors, and regulators [15]. Differential expression analysis identifies hormone-responsive modules that coordinate multi-stress responses; co-expression network analysis (WGCNA) links gene clusters to stress-tolerance traits such as osmotic adjustment, ROS scavenging, and membrane stability [16].

Time-series transcriptomics captures the temporal dynamics of hormonal crosstalk during stress progression and recovery, distinguishing early alarm signals from long-term acclimation responses [17]. Spatial transcriptomics, including laser-capture microdissection, RNA-seq, and in situ sequencing, maps cell-type-specific expression gradients across root endodermis, leaf mesophyll, and guard cells, revealing microscale compartmentalization of ABA, ethylene, and jasmonate signaling under stress [18]. Single-cell RNA-seq has identified heterogeneous stress responses within seemingly uniform tissues, uncovering rare cell populations with enhanced stress tolerance signatures. Functional transcriptomics employs eQTL mapping to link genetic variants to stress-responsive expression plasticity and TWAS to link gene expression variation to field-measured stress-tolerance phenotypes [19].

Recent transcriptomic profiling under drought, salinity, and temperature stress has revealed coordinated expression modules linking ABA, ethylene, and jasmonic acid signaling, and multi-omics integration has identified master regulators that orchestrate cross-pathway crosstalk (Table 1) [17].

### 2.2. Proteomics and Phosphoproteomics: Revealing Post-Translational Regulation

Proteomics elucidates hormonal stress pathways by uncovering changes in protein abundance, post-translational modifications (PTMs), and protein interactions under abiotic stress (Table 1; Figure 1) [14]. High-resolution mass spectrometry with isobaric tagging (iTRAQ, TMT) measures global protein profiles across stressed tissues, identifying ABA receptors, MAPK cascades, LEA proteins, and stress-induced transporters [20]. Phosphorylation is central to rapid stress signaling, regulating ABA-mediated stomatal closure, osmotic adjustment, and stress kinase activation within minutes [21]. Phosphoproteomics has mapped thousands of stress-regulated sites on SnRK2 kinases and PP2C phosphatases, revealing temporal hierarchies in drought and salinity networks [22]. Ubiquitinome profiling uncovers protein turnover that controls hormone receptor stability and stress memory, while acetylation and sumoylation modulate transcription factor activity during prolonged stress [20]. Spatial proteomics examines protein dynamics across root zones and leaf cell types under heat or cold stress, revealing tissue-specific hormone signatures. Mapping stress-induced protein complexes through affinity purification-mass spectrometry defines how ABA, ethylene, and jasmonate signaling machinery assembles under stress (Table 2) [23].

### 2.3. Metabolomics: Quantifying Hormone Levels and Metabolic Shifts

Metabolomic techniques reveal biochemical states that regulate hormonal stress signaling in plants, identifying small-molecule intermediates, hormone gradients, and crosstalk metabolites that govern stress responses (Table 1; Figure 1) [24]. Techniques such as mass spectrometry (GC-MS, LC-MS/MS) and NMR spectroscopy measure stress-responsive metabolites, including ABA conjugates, jasmonic acid derivatives, auxin metabolites, compatible solutes (proline, glycine betaine), antioxidants (ascorbate, glutathione), and membrane lipid products [25,26]. These techniques map hormone dynamics during drought, cold acclimation, salinity adaptation, and heat stress. Targeted metabolomics enables precise quantification of specific hormones and biosynthetic intermediates, revealing tissue-specific hormone pools responding rapidly to environmental cues [27]. Untargeted metabolomics detects broad metabolic reprogramming, identifying crosstalk metabolites like oxylipins, flavonoids, raffinose oligosaccharides, and amino acid derivatives that integrate hormonal pathways with primary and secondary metabolism [27,28]. These approaches reveal regulatory nodes in which metabolite signatures precede transcriptional changes, thereby defining early stress-perception events and metabolic checkpoints that control stress-tolerance thresholds [27]. Integration of hormone measurements with metabolic flux analysis quantifies pathway activity under stress conditions, thereby distinguishing biosynthesis, transport, and degradation rates. Recent applications of metabolomic techniques in forest hormone systems are summarized in Table 2.

### 2.4. Epigenomics: Heritable Regulation of Hormone Signaling

Epigenomic mechanisms modulate hormone-responsive gene expression and establish stress memory in forest trees (Table 1; Figure 1). Techniques such as whole-genome bisulfite sequencing (WGBS) map DNA methylation at single-base resolution, revealing dynamic changes in DNA methylation at ABA-responsive promoters during drought [29]. Reduced-representation bisulfite sequencing (RRBS) enables cost-effective methylome profiling, while long-read platforms simultaneously resolve methylation and structural variants in large genomes [29]. These techniques reveal epigenetic reprogramming during cold acclimation, heat memory, and multi-generational drought adaptation. Chromatin immunoprecipitation sequencing (ChIP-seq) identifies active (H3K4me3) and repressive (H3K27me3) histone marks regulating ABA biosynthesis genes and stress transcription factors [30]. CUT&RUN and CUT&TAG offer superior sensitivity in limited tissue samples, mapping chromatin states that control rapid stress-induced transcriptional reprogramming and establish long-term epigenetic memory [31,32]. Small RNA sequencing identifies stress-responsive microRNAs post-transcriptionally regulating hormone receptors under abiotic stress [31,32]. Integration with transcriptomics reveals how chromatin modifications gate activation of hormone pathways, establishing molecular memory that primes plants for recurring stress episodes and enables transgenerational stress adaptation. Recent applications of epigenomic techniques in forest hormone systems are summarized in Table 2.

**Table 2:** Multi-omics applications across diverse forest and woody species.

Species	Tissue/Organ	Functional Role	Mutli-omics	Hormones Included	Ref.
*Salix caprea*	Roots	Cadmium toxicity with Phosphorus treatment	Physiology, Transcriptomics, and Metabolomics	Auxin and ABA	[33]
*Maclura tricuspidata*	Roots and leaves	Salt stress	Physiology, Transcriptomics, and Metabolomics	ABA, auxin, ethylene	[34]
*Quercus fabri*	Axillary buds	Branching development, wood yield, and vegetative growth	Transcriptomics, Proteomics, and Metabolomics	IAA (auxin), trans-zeatin; ABA, ACC JA	[35]
*Eucalyptus urophylla × Eucalyptus grandis*	Immature xylem	Wood formation, secondary cell wall biosynthesis, lignin deposition, and fiber development	Proteomics	Auxin (IAA), Cytokinin, GA, ABA, Ethylene	[36]
*Quercus fabri* Hance	Axillary buds	Axillary bud, sucrose anabolic pathway; branching and tree architecture	Transcriptomics, Proteomics	Auxin (IAA), Cytokinin, ABA, ACC, JA	[37]
*Eucalyptus urophylla × Eucalyptus grandis*	Vascular cambium	Secondary growth, wood formation, and age-dependent cambial activity	Transcriptomics (RNA-seq profiling)	Auxin, Cytokinin, GA, ABA (ABA), Ethylene	[38]
*Hybrid Liriodendron* (e.g., *Liriodendron chinense × Liriodendron tulipifera*)	Axillary buds	Axillary bud development; branching control	Transcriptomics (RNA-seq) + Functional validation (gene expression assays, transgenic/KD approaches)	Auxin, Cytokinin, ABA, ACC, JA	[39]
*P. trichocarpa*	Stem-differentiating xylem, cambium, buds	Abiotic stress: drought, cold, heat Growth: xylem development	Epitranscriptomics and proteomics	ABA, auxin, cytokinin, ethylene, GA	[40]
*Populus × canescens*	xylem and cambium	Severe drought	Transcriptomics, targeted hormone profiling	ABA, auxin, cytokinin, jasmonic acid, salicylic acid	[41]

### 2.5. Multi-Omics Perspectives on Hormone-Mediated Stress Responses

Multi-omics integration has transformed understanding of hormone-mediated stress responses in forest species by revealing temporal regulatory hierarchies, cross-pathway interactions, and heritable adaptive mechanisms that single-omics approaches cannot resolve (Table 2). These integrated analyses distinguish rapid post-translational responses (minutes to hours) from slower transcriptional and metabolic reprogramming (hours to days), identifying regulatory bottlenecks and master coordinators of multi-stress tolerance [42].

In Populus under progressive drought, phosphoproteomic profiling revealed SnRK2 kinase activation and aquaporin phosphorylation within 2 h of water-deficit onset, preceding transcriptional responses by 6–12 h [43]. Subsequent transcriptomic analysis showed coordinated upregulation of ABA biosynthesis genes (NCED3, AAO3) and stress-responsive transcription factors (ABF2/AREB1, DREB2A, NAC family members), while metabolomic profiling detected accumulation of proline, glycine betaine, and flavonoid antioxidants after 24 h [43]. This temporal hierarchy establishes that post-translational modification of pre-existing signaling components constitutes the primary rapid stress response, enabling immediate stomatal closure and osmotic adjustment before transcriptional reinforcement. Integration of proteomics and transcriptomics further revealed that downregulation of photosynthetic proteins under combined drought-salinity stress occurs through both reduced transcript abundance and enhanced protein degradation, with components of the ubiquitin–proteasome pathway upregulated at the protein level despite unchanged mRNA levels [44].

Phosphoproteomic analysis of poplar roots revealed crosstalk between brassinosteroid and auxin pathways under nitrogen stress, with shared targets such as receptor kinases (BRI1, TIR1) and transcription factors [45,46]. This integration coordinates root responses: brassinosteroids promote lateral root elongation, while auxin redistribution specifies initiation sites, optimizing nitrogen foraging under nutrient limitation. In *Salix caprea* roots exposed to cadmium toxicity under phosphorus deficiency, integrated transcriptomic and metabolomic analyses revealed that the auxin and ABA pathways converge to regulate metallothionein expression and phytochelatin biosynthesis, with auxin-responsive ARF genes and ABA-responsive ABF genes co-regulating detoxification enzymes and root developmental remodeling [47].

Whole-genome bisulfite sequencing in Populus revealed stable DNA methylation changes at genes in the auxin and gibberellin pathways that persisted across seasons. These epigenetic marks established epigenetic stress memory: cold-primed trees exhibited faster induction of cold-responsive genes and enhanced freezing tolerance in subsequent winters than naïve trees, despite identical genotypes [48]. Small RNA sequencing revealed that stress-responsive microRNAs (miR160 and miR167 targeting ARF genes; miR319 targeting TCP transcription factors) accumulate during initial stress exposure and maintain elevated expression through epigenetic reinforcement of MIR gene promoters [44]. Long non-coding RNAs (lncRNAs) further modulate stress memory: drought-responsive lncRNAs antisense to ABA biosynthesis genes (NCED-AS, ABA2-AS) stabilize stress-induced transcript levels and accelerate ABA accumulation during repeated drought episodes [44,49]. In *Maclura tricuspidata*, transcriptomic and metabolomic integration demonstrated that ABA, auxin, and ethylene pathways coordinate distinct tissue-specific responses: ABA signaling dominated leaf responses (stomatal regulation, osmolyte synthesis), auxin redistribution restructured root architecture, and ethylene signaling modulated root aerenchyma formation, collectively optimizing water and nutrient acquisition under salt stress [34].

These studies identify targets to improve stress resilience in forests. Understanding hormone crosstalk guides strategies: enhancing brassinosteroid and auxin signaling boosts the nutrient stress response, while balancing jasmonate and ABA signaling enhances pathogen resistance without yield loss under drought. More examples across forest genera are in Table 2.

## 3. Limitations of Traditional Omics Integration in Forest Hormone Biology

### 3.1. Biological Limitations in Forest Trees

#### 3.1.1. Data Heterogeneity and Tissue Complexity

Multi-omics layers differ markedly in structure, dynamic range, and noise, creating substantial barriers to integrated analysis in forest stress-response research [50]. Thus, transcriptomic datasets provide normalized gene expression profiles, whereas proteomic data often contain missing values, peptide redundancies, and narrower quantitative ranges. For instance, metabolomic profiles fluctuate strongly across environments, developmental stages, and tissue types, a particular concern for stress-induced hormones whose abundance changes rapidly under drought, heat, nutrient limitation, or pathogen attack. Meanwhile, epigenomic datasets add further complexity, as DNA methylation and histone modifications vary across cell types and seasons, particularly during stress-acclimation cycles in perennial species [51]. These disparities necessitate extensive preprocessing and normalization, yet even after correction, cross-omics correlation remains limited in many forest species due to incomplete or poorly annotated reference genomes.

A major biological constraint arises from the reliance on bulk tissue sampling, which collapses the inherent spatial heterogeneity of forest organs. Hormone-mediated stress responses are highly localized: abscisic acid accumulates in guard cells to regulate stomatal closure during drought; ethylene gradients at root tips modulate growth under compaction or flooding; jasmonate bursts occur in wounded or pathogen-challenged tissues [52,53]. Such microdomain signaling is effectively erased when tissues are homogenized. In forest trees, this problem is amplified by the anatomical complexity of stems, roots, and perennial structures. Bulk sampling merges signals from xylem, phloem, cambium, cortex, bark, and associated tissues, each with distinct hormonal sensitivities and stress-response roles [52]. As a result, subtle hormone gradients are flattened, cell-specific transcriptional programs are diluted, and transient stress-induced metabolites are masked by dominant tissue signals. This loss of spatial resolution severely restricts the interpretability of multi-omics datasets and limits the reconstruction of accurate hormone-mediated regulatory networks under stress [52].

Temporal limitations further constrain traditional omics integration. Hormone signaling in forest species is tightly coupled to environmental fluctuations, drought pulses, heat waves, nutrient shifts, and pathogen invasion, yet most multi-omics studies rely on single or sparsely spaced time points. These static snapshots fail to capture the rapid, transient hormone pulses that initiate stress responses, many of which occur within minutes to hours [54,55,56]. Without high-resolution time-series data, key features of stress-induced hormonal regulation, including oscillatory dynamics, feedback loops, and crosstalk among ABA, ethylene, jasmonate, and salicylic acid pathways, remain unresolved. Consequently, the reconstruction of hormone signaling trajectories is incomplete, narrowing the interpretive power of multi-omics studies and limiting our understanding of how hormonal networks orchestrate stress adaptation in forest species [57].

#### 3.1.2. Tissue Complexity and Sampling Challenges in Forest Trees

Forest trees possess highly integrated and structurally complex organs, making it difficult to obtain clean, cell type-specific samples for multi-omics analyses [58]. Woody stems, perennial shoots, and deep root systems contain tightly interwoven tissues: xylem, phloem, cambium, cortex, bark, and associated parenchyma that cannot be easily separated without cross-contamination [59]. As a result, even carefully executed sampling procedures often yield mixed tissue populations. This poses a major limitation for hormone-mediated stress biology, where regulatory activity is frequently confined to narrow spatial domains such as the cambial zone during drought, root tips under compaction stress, or wound-activated cells during herbivory [60]. When these microdomains are blended into bulk samples, subtle hormone gradients are obscured, cell-specific transcriptional responses are diluted, and the resulting multi-omics profiles fail to reflect the true spatial organization of stress signaling networks [60].

Sampling challenges extend beyond anatomical heterogeneity. Many hormone-related metabolites central to stress responses are chemically unstable and degrade rapidly during collection, transport, or extraction. Compounds such as indole-3-acetic acid (IAA), jasmonates, and reactive oxygen species can fluctuate within minutes of wounding, oxygen exposure, or temperature shifts [61]. In forest tree organs, where hormone gradients are often subtle, transient, and spatially restricted, such degradation can severely compromise data reliability and distort interpretations of stress-induced metabolic dynamics [61].

Together, these anatomical and biochemical constraints limit the precision of traditional multi-omics integration in forest species. Consequently, multi-omics datasets often provide only coarse approximations of stress-regulated processes that are, in reality, highly organized, cell type-specific, and spatially intricate [62].

#### 3.1.3. Temporal Limitations: Static Snapshots of Dynamic Processes

Hormone signaling in forest trees is dynamic, characterized by rapid shifts in hormone levels, receptor sensitivities, and gene activity in response to environmental cues such as drought, temperature, nutrient availability, and pathogens. However, most multi-omics studies use limited sampling, providing static snapshots that miss the true temporal complexity of stress responses. This overlooks key regulatory details such as timing, intensity, and sequence of hormone-driven events [54,55].

Transient hormone pulses, often lasting only minutes to hours, play decisive roles in initiating stress acclimation or developmental transitions. These include ABA spikes during early drought perception, jasmonate bursts after herbivory, and ethylene surges under hypoxia. Such short-lived signals are easily missed in coarse sampling schemes. Similarly, feedback loops between hormone pathways, such as auxin–cytokinin antagonism, and seasonal regulatory transitions, such as dormancy cycling, depend on tightly phased temporal dynamics that cannot be reconstructed from isolated time points [56].

In perennial forest species, where stress responses unfold across multiple temporal scales, from minutes during acute stress to months across seasonal cycles, the consequences of these limitations are especially pronounced [63]. Without high-resolution time-series data, integrative models risk oversimplifying regulatory networks, misidentifying causal relationships, or failing to detect critical crosstalk among ABA, ethylene, jasmonate, salicylic acid, and other stress-responsive pathways. Ultimately, the inability to resolve these fluctuations limits the reconstruction of accurate hormone signaling trajectories, narrowing the interpretive power of multi-omics studies and constraining our understanding of how hormonal regulation orchestrates stress adaptation in forest species [57].

### 3.2. Computational and Methodological Limitations

#### 3.2.1. Reductionist Integration Methods Miss Nonlinear Regulatory Interactions

Traditional multi-omics integration approaches can detect broad co-expression patterns and enriched pathways, yet they remain poorly suited to resolving the nonlinear, multilayered interactions that define hormone-mediated stress responses in forest species [64]. Hormone networks rarely act independently; instead, they exhibit antagonistic, synergistic, and context-dependent crosstalk that shifts dynamically in response to drought, temperature extremes, nutrient imbalances, or pathogen challenges. Linear statistical models, still common in many integration pipelines, assume proportionality and independence among variables, preventing them from capturing threshold effects, feedback loops, and emergent regulatory behaviors central to stress-induced hormone signaling [65]. Consequently, these methods often yield descriptive correlations rather than mechanistic insights, limiting the ability to infer causal interactions or identify regulatory motifs that orchestrate rapid hormonal adjustments during stress [66]. Computational constraints further restrict multi-omics integration in forest systems. Despite advances in long-read sequencing, many forest genomes remain fragmented, highly repetitive, and heterozygous, complicating genome assembly and annotation [67]. These limitations propagate into downstream analyses: incomplete gene models reduce the accuracy of transcript, protein, and metabolite mapping, thereby weakening confidence in inferred hormone-responsive pathways. Integrating datasets across tissues, developmental stages, and diverse stress conditions also imposes substantial computational demands. High-dimensional omics layers require sophisticated algorithms for normalization, batch correction, network inference, and statistical modeling, yet many available tools were developed for small, well-annotated genomes such as *Arabidopsis thaliana* and do not scale effectively to the complexity of forest species [67,68].

Annotation gaps remain a major bottleneck, particularly in proteomics and metabolomics. Unidentified proteins, ambiguous peptide assignments, and large pools of unknown or poorly characterized metabolites limit the interpretability of integrated datasets [69]. These deficiencies hinder the reconstruction of comprehensive hormone signaling networks and reduce the reliability of models seeking to explain how forest trees perceive, integrate, and respond to environmental stress.

#### 3.2.2. Limited Transferability Across Species and Ecological Contexts

Most multi-omics frameworks are developed using annual crops or model species, whose physiology, life history, and ecological strategies differ substantially from those of forest trees. As a result, models trained on herbaceous plants often perform poorly when applied to forest systems, where hormone-mediated stress responses unfold over long lifespans and are shaped by chronic acclimation, seasonal variability, and complex biotic interactions [70,71]. Forest species often have unique gene families, hormonal sensitivities, and long-term regulatory programs, which pose challenges for many existing integration tools. This limits their ability to accurately capture species-specific regulatory responses during drought, stress, or pathogen attack. As a result, cross-taxa extrapolation is unreliable, and the lack of forest-specific frameworks risks oversimplifying or misrepresenting stress responses in trees [72].

The challenge is compounded by the complexity of multi-omics datasets, which contain thousands of variables across molecular layers. Conventional visualization approaches, such as heatmaps, correlation matrices, and static network diagrams, struggle to represent the temporal dynamics, conditional interactions, and cross-layer regulatory shifts that define hormone signaling under stress [51,73]. These static outputs often obscure rather than clarify key regulatory patterns, making it difficult for non-specialist audiences, including forest managers, policymakers, and interdisciplinary collaborators, to interpret multi-omics findings. When visualizations fail to convey mechanistic insights or actionable patterns, the translation of multi-omics research into forest management, conservation planning, or stress-resilient breeding strategies becomes severely constrained [68].

### 3.3. AI-Augmented Multi-Omics as a Complementary Framework in Forest Hormone Biology

The constraints in Section 3.1 and Section 3.2 show that traditional multi-omics remains vital for forest hormone biology, but its interpretive power is limited (Table 3). This does not diminish traditional methods but underscores the need for tools like AI-augmented multi-omics, which can extract deeper insights from complex data.

Traditional methods provide biologically grounded, transparent, and experimentally validated insights, whereas AI extends these foundations by enabling automated fusion of heterogeneous omics layers, modeling nonlinear hormone crosstalk, and predicting stress outcomes beyond descriptive enrichment analyses [54] (Table 3). Machine learning and deep learning frameworks, Bayesian networks, graph neural networks, and recurrent architectures capture dynamic, context-dependent regulatory interactions that conventional correlation-based networks cannot resolve [74]. AI also scales effectively to multi-tissue and multi-genotype datasets and supports predictive modeling of combined drought–heat–nutrient stresses, which are increasingly relevant under climate change [74].

However, AI cannot replace traditional omics, particularly in forest systems where datasets are small, heterogeneous, and sparsely annotated. Deep learning models risk overfitting and often produce latent features that are difficult to map onto known hormone pathways [75]. Explainable AI (XAI) frameworks, SHAP, Integrated Gradients, attention mechanisms, sparse autoencoders, and hybrid pipelines are therefore essential for ensuring biological interpretability and maintaining mechanistic relevance (Table 3) [75]. Strategies such as transfer learning, self-supervised learning, graph-based modeling, and dimensionality reduction further mitigate data scarcity [74,75].

Together, traditional and AI-augmented multi-omics form a synergistic framework: traditional omics anchors analyses in biological reality, while AI enhances integration, prediction, and systems-level inference. This hybrid approach offers a more complete reconstruction of hormone-mediated stress networks and supports translational applications in climate-resilient forestry.

## 4. Integrating AI and Traditional Multi-Omics Approaches for Hormone-Mediated Abiotic Stress Responses

**Figure 2:**
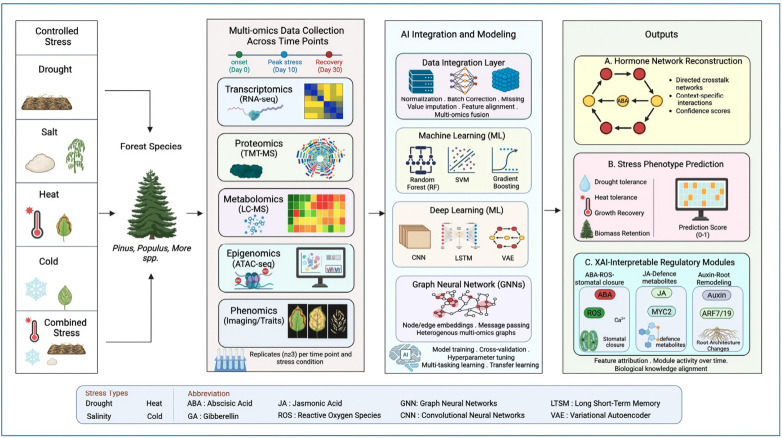
AI-augmented multi-omics framework for decoding hormone-mediated stress responses in forest species. Schematic overview of an integrative experimental and computational pipeline combining controlled abiotic stress treatments (drought, salinity, heat, cold, and combined stress) with multi-omics profiling in forest tree species such as *Pinus* and *Populus* spp. Time-resolved sampling across onset, peak stress, and recovery stages captures transcriptomic (RNA-seq), proteomic (TMT-MS), metabolomic (LC-MS), epigenomic (ATAC-seq), and phenomic traits (imaging-based measurements). AI-driven data integration incorporates normalization, batch correction, feature alignment, and multi-omics fusion, followed by machine learning (RF, SVM, gradient boosting), deep learning (CNN, LSTM, VAE), and graph neural networks (GNNs) for network inference and prediction. Model outputs include (A) reconstruction of hormone-centered regulatory networks, (B) prediction of stress-tolerance phenotypes such as drought or heat resilience, and (C) interpretable regulatory modules (XAI) highlighting key pathways including ABA–ROS–stomatal closure, JA-linked defense metabolism, and auxin-mediated root remodeling. This framework enables mechanistic insight and predictive modeling of hormone-regulated stress adaptation in forest systems. This figure was drawn using Bio render online software (https://app.biorender.com/).

AI–traditional multi-omics integration provides a unified analytical framework that leverages the strengths of both established statistical methods and advanced machine-learning architectures. Traditional approaches, including differential expression analysis, WGCNA, clustering, and pathway enrichment, remain essential for generating mechanistically interpretable regulatory modules across transcriptomic, proteomic, and metabolomic layers (Table 3). AI-driven models such as GNNs, transformers, and PLMs extend this foundation by resolving nonlinear regulatory interactions, modeling high-dimensional dependencies, and predicting emergent phenotypes that conventional methods alone cannot capture (Figure 2; Table 4) [76,77].

In hormone-mediated stress biology, the regulatory landscape is shaped by the type, duration, and combination of abiotic cues. Crosstalk nodes such as the ABA–ROS–stomatal closure axis during drought, ABA–ethylene antagonism under flooding, and JA–SA trade-offs during combined stress represent multilayered intersections that require integrative analysis [78]. Traditional multi-omics provides statistical confidence and mechanistic grounding for these pathways, while AI models infer directionality, temporal dynamics, and context-dependent modulation of hormone signaling from complex datasets [79,80]. Together, these complementary approaches enable a more comprehensive reconstruction of hormone-regulated stress networks, supporting the development of testable hypotheses and predictive models for abiotic stress resilience in forest systems.

### 4.1. Machine Learning Approaches for Multi-Omics Integration in Abiotic Stress Contexts

Random Forests, gradient boosting, and regression-based ML models provide predictive frameworks linking hormone signaling to phenotypic outcomes. They capture non-linear, hierarchical hormonal stress interactions, feedback, thresholds, and crosstalk without needing explicit mechanistic assumptions [81] (Figure 2; Table 4).

#### 4.1.1. Random Forest

Random Forest (RF) models are widely used in plant systems biology because they tolerate noise and missing values and capture nonlinear feature interactions, making them well suited for high-dimensional multi-omics datasets generated under abiotic stress (Table 4) [82,83]. In *Pinus taeda*, metabolomics-based RF-ML revealed ABA- and SA-linked osmotic adjustment and antioxidant reprogramming under long-term drought [84]. In drought studies, RF classifiers have been applied to identify ABA-responsive transcriptional signatures associated with stomatal regulation and osmotic adjustment, to resolve regulatory modules that are difficult to detect using single-omics analyses alone [85]. Rico-Chávez et al. [86] further demonstrated how ML approaches, including ANN, SVM, and RF, capture hormetic dose–response patterns and proposed an ABA- and JA-centered framework for improving stress tolerance in forest tree breeding. Under salinity stress, RF models have been used to predict metabolite accumulation associated with hormone-mediated osmotic adjustment, revealing JA- and ABA-regulated biosynthetic pathways linked to adaptive traits [87]. Xiong et al. [35] applied RF-integrated multi-omics in *Quercus fabri* to dissect branching regulation, identifying auxin–cytokinin signaling components as key modulators and illustrating how drought-induced ABA accumulation reprograms auxin transport and root architectural responses in tree species.

In addition, RF-ML models have demonstrated consistent value for classifying stress responses, identifying biomarkers, and uncovering regulatory signatures across molecular layers [88,89]. For instance, association genetics integrated with transcriptomics in Populus demonstrated that eQTLs controlling ABA- and auxin-responsive modules predict drought tolerance, with 1413 DEGs linked to genomic variants [90]. These results highlight the potential of AI-augmented multi-omics to accelerate breeding for hormone-mediated resilience traits. Additional research across multiple species, including forest species, using ML-based hormesis modeling, demonstrated that sublethal stress doses induce beneficial ABA- and JA-mediated acclimation responses [86], providing a quantitative basis for stress-priming strategies in forestry.

Overall, RF models bridge data-driven inference and experimental validation, helping identify hormone-regulated nodes for functional studies. Additional AI-augmented multi-omics examples in forest hormonal biology are summarized in Table 5.

#### 4.1.2. Support Vector Machines, Gradient Boosting, and Ensemble Models

Support Vector Machines (SVMs) are particularly effective in plant and forest systems biology, where sample sizes are often limited due to long generation times and constraints in controlled abiotic stress experiments [88]. In hormone-centered stress biology, SVMs classify hormone-responsive transcriptional modules and discriminate stress severity using ABA- or JA-linked metabolic signatures, offering a quantitative framework for drought, heat, and salinity responses (Table 4) [88]. A meta-analysis by Tahmasebi et al. [91] combined SVMs, Random Forest, and WGCNA to identify water stress-responsive genes in *Populus* spp., revealing MYB and MAPK hub genes and strong transcriptional shifts in ABA- and SA-related pathways. Tissue-specific SVM classification has further refined the spatial resolution of hormone biosynthesis, identifying zones of ABA or JA production in guard cells, cambial tissues, and phloem, providing insights that extend beyond what bulk transcriptomics can resolve [92].

SVMs excel at defining decision boundaries in complex, nonlinear datasets, ideal for distinguishing subtle transitions in hormone-responsive states. They differentiate gradual ABA signaling during drought from rapid heat stress activation. Reviews show that SVM tools achieve 60–78% accuracy in predicting stress-responsive genes and emphasize the need for explainable AI to identify regulatory genes across multi-omics datasets for drought, heat, cold, and salinity [76]. Additional examples of SVM multi-omics applications in forest hormonal biology are summarized in Table 5.

**Table 5:** Summary of AI-augmented Applications in Forest Hormone Biology Systems.

Forest Species	Hormone/Omics Layers	AI Method	Stress: Key Findings	Ref.
*Populus* spp.	ABA, salicylic acid/Transcriptomics (RNA-seq)	Meta-analysis, ML (Random Forest, SVM), Systems biology/WGCNA	Drought: MYB and MAPK hub genes; ABA-dependent and SA-responsive cis-elements; putative cytochrome P450.	[91]
*Populus balsamifera*	ABA/Epigenomics (whole-genome bisulfite sequencing)	Deep Learning (multilayer artificial neural networks)	Abiotic stress: DNA methylomes; epigenetic variation captures stress-adaptive traits.	[93]
*Pinus tabuliformis*	ABA/Transcriptomics (RNA-seq)	WGCNA (co-expression network), hub gene analysis	Drought: 10 network hub genes integrating ABA-dependent and ABA-independent pathways; PtNCED3 expression under drought; 84 drought-TFs and 62 protein kinases.	[94]
*Pinus taeda*	ABA, SA, GA (GA4)/Metabolomics (targeted and untargeted)	Multivariate statistical analysis (PCA, PLS-DA), pathway enrichment	Drought: ABA and SA under long-term drought (42 d); GA4 increased in roots.	[84]
*Juglans regia*	MeJA/Transcriptomics + Metabolomics	Transcriptome–metabolome correlation network analysis, pathway enrichment	Salinity: MeJA-mediated induction of phenylalanine and antioxidant biosynthesis pathways; JA signaling to enhance ROS scavenging capacity.	[95]
Multiple plant species	ABA, JA, ethylene/miRNA omics/small RNA sequencing + sequence feature databases	GNN/graph autoencoder based on Graph Isomorphism Networks; Restart Random Walk algorithm	Abiotic stresses: GNN achieved AUPR 98.24% and AUC 97.43% (5-fold CV) for predicting miRNA; miR168, miR393, and miR396 highlighted as cross-stress regulatory nodes linked to hormone signaling.	[96]
*Pinus massoniana*	ABA, ethylene (AP2/ERF TF)/Transcriptomics (RNA-seq)	DEG analysis, WGCNA, functional enrichment	Drought: AP2/ERF1 TF; SOD activity and MDA levels used as physiological validation markers.	[97]
*Populus* spp.	ABA, auxin/Transcriptomics + Association genetics (RNA-seq + SNP genotyping)	eQTL mapping, co-expression network analysis, association genetics	Drought: eQTL analysis linked 1413 drought-responsive DEGs to genomic variants; candidate genes for ABA-mediated drought tolerance validated by epistasis analysis.	[90]
*Pinus tabuliformis*	ABA, JA/Transcriptomics + Metabolomics	Gene–metabolite correlation network, WGCNA, KEGG/GO enrichment	Drought: JA and proline metabolic networks; ABA signaling (PYR/PYL-PP2C-SnRK2) and JA biosynthesis (AOC, AOS) co-regulated under water deficit.	[98]
Multiple plant species	ABA, JA/Transcriptomics, Physiomics	ML (regression, ANN, SVM, Random Forest); hormesis dose–response modeling	Abiotic stresses: Non-linear hormetic dose–stress responses; sublethal stress doses linked to positive ABA- and JA-mediated acclimation outcomes; framework applicable to forNest tree stress tolerance breeding.	[86]
*Pinus massoniana*	ABA, JA/Transcriptomics + Metabolomics	DEG analysis, KEGG metabolic pathway enrichment, transcriptome–metabolome integration	Drought + Heat: Starch/sucrose metabolism genes differentially expressed; ABA and JA signal transduction pathways activated; key metabolites in photosynthesis–carbon coupling identified.	[99]

Gradient-boosting algorithms, including XGBoost, LightGBM, and CatBoost, combine weak learners into ensembles to capture complex, nonlinear interactions across multi-omics layers, consistently achieving high predictive accuracy in plant systems biology (Figure 2; Table 4) [80,100]. In hormone-mediated abiotic stress research, boosting models have been used to predict ABA, JA, and GA levels directly from transcriptomic and proteomic features, enabling inference of hormone dynamics when metabolomic datasets are incomplete or unavailable [101]. Chen et al. [102] used a kernel-augmented neural boosting model (KANMB) to analyze transcriptomic and metabolomic data in salt-stressed plants, revealing JA- and ABA-related metabolic modules and key gene–metabolite interactions in the hormonal regulation of salt tolerance. Indicating that boosting architectures can identify hormone-linked regulatory signatures from multi-omics data, even with limited or no direct hormone measurements. Mehdizadeh Hakkak and Tohidfar [103] used XGBoost in a transcriptomic framework, showing that boosting classifiers outperform traditional models for identifying abiotic stress biomarkers in Arabidopsis. Many of the top features were hormone-responsive genes, indicating that gradient boosting captures hormone-linked transcriptional signatures associated with stress resilience. While little work has been done on forest species, these technologies could enhance boosting models for inferring hormone-associated regulatory states in forest systems.

### 4.2. Deep Learning for Multi-Omics Integration

Deep learning models learn hierarchical, nonlinear representations from large, complex datasets, automatically extracting multiscale patterns from diverse data sources without manual feature engineering [104]. In plant hormone biology, DL is particularly valuable for multi-omics datasets spanning molecular, anatomical, and environmental layers, as well asfor regulatory processes that unfold across spatial and temporal dimensions that ML models cannot capture at the same resolution (Figure 2; Table 4). The growing availability of multi-omics data from plant abiotic stress experiments, covering transcriptomic, proteomic, and metabolomic profiles over time, offers opportunities for DL models to uncover regulatory structures underlying hormone-mediated adaptation [77,104].

#### 4.2.1. Convolutional Neural Networks, Recurrent Neural Networks, and Long Short-Term Memory Models

Convolutional Neural Networks (CNNs) excel at spatial inference and are ideal for studying hormone gradients and tissue organization under abiotic stress. Liu et al. [105] used single-cell RNA-seq and spatial transcriptomics to show that ABA from phloem cells drives cell-specific gene changes in response to soil compaction. Demonstrating how spatial datasets, analyzed with CNNs and other algorithms, can resolve hormone gradients within tissues at resolutions beyond bulk omics. CNNs can also infer auxin and cytokinin gradients from spatially resolved transcriptomic or reporter imaging datasets, revealing microdomains of hormonal activity that govern cell fate decisions under stress [7]. Earlier, Champigny et al. [93] applied deep learning to whole-genome methylomes of *Populus balsamifera*, explaining 57.5% of biomass variance and 40.9% of wood-density variance from natural DNA methylation patterns, illustrating how epigenomic variation captured by AI models reflects stress-adaptive phenotypes in forest populations. CNNs applied to hyperspectral or UAV imagery further enable phenotype prediction at landscape scales, linking canopy reflectance patterns, influenced by drought-induced, ABA-mediated stomatal closure, to hormone-regulated processes [106,107]. Such approaches are particularly relevant to forest stress biology, where spatial heterogeneity and environmental gradients across canopy positions and stand structures yield hormonal responses that are invisible to traditional sampling designs.

Temporal modeling is vital for understanding hormone regulation in response to evolving abiotic stresses over timescales ranging from hours to years. Recurrent Neural Networks (RNNs) and Long Short-Term Memory (LSTM) architectures are designed to capture sequential dependencies in time-series data, making them suitable for modeling the phased hormonal responses characteristic of drought progression, cold acclimation, or heat-stress recovery [108]. In plant hormone biology, LSTM models trained on time-series transcriptomic and metabolomic data learn the sequence of ABA accumulation, TF activation (DREB, MYB, NAC), osmolyte biosynthesis, and growth recovery, capturing regulatory dynamics that static models cannot resolve. Applied to combined-stress scenarios, LSTMs model the sequential activation of ABA–ethylene crosstalk during flooding followed by drought, or the phased induction of JA-SA antagonism under heat waves superimposed on pathogen pressure [89]. A particularly important application in perennial systems is modeling hormone dynamics across seasonal developmental transitions, dormancy induction, bud break, and growth cessation, using increasingly available multi-year transcriptomic datasets for temperate tree species [109]. Examples are summarized in Table 5.

#### 4.2.2. Autoencoders and Variational Autoencoders

Deep autoencoders and variational autoencoders (VAEs) provide compressed representations that reveal hormone-specific response states not visible in raw omics data. They reduce dimensionality, integrate multi-omics data under stress conditions, and preserve key regulatory structure while filtering out noise [110]. This is critical for datasets that combine transcriptomic, proteomic, metabolomic, and epigenomic layers across various stress conditions. Their utility is particularly evident in epigenomic datasets, where high dimensionality and sparse regulatory signals hinder classical analyses. Earlier, In *Populus balsamifera*, multilayer neural networks trained on whole-genome methylomes explained 57.5% of biomass variance and 40.9% of wood density variance, demonstrating that epigenetic variation, much of it being ABA-linked, encodes stress-adaptive phenotypes across environments [93].

While variational autoencoders (VAEs) extend this framework by learning probabilistic latent spaces, they enable the modeling of biological variability and the generation of synthetic omics profiles [8,111]. Zhao et al. [112] demonstrated the utility of multiscale VAEs for imputing missing values in untargeted metabolomics datasets using genomic sequence data, a capability directly applicable to hormone metabolite profiling under abiotic stress, where data completeness varies across species and experimental conditions. VAEs also enable data augmentation by generating synthetic training samples, addressing one of the most persistent constraints on DL applications in abiotic stress research: the limited sample sizes that characterize most controlled stress experiments, in which the cost and complexity of multi-omics profiling limit replication. [112].

Transformers excel at modeling sequential and contextual information, making them ideal for time-series omics that capture ABA, JA, and ethylene signaling dynamics under drought, heat, and combined stresses. Their ability to integrate transcriptomic, proteomic, and chromatin accessibility data enables the reconstruction of delayed hormone responses and the prediction of stress-response trajectories. This aligns with empirical findings in *Pinus massoniana*, where drought–heat co-stress induced non-additive ABA–JA signaling and reprogrammed carbon metabolism [99]. Dynamic modeling frameworks further support the prediction of stomatal behavior, ABA–ROS oscillations, and JA-mediated defense activation (Table 5), providing a quantitative basis for forecasting stress adaptation in forest ecosystems.

### 4.3. Graph Neural Networks: Reconstructing Hormone Signaling Networks Under Stress

Hormone signaling pathways are graph-structured systems. Abiotic stresses disrupt these networks, and reconstructing stress-induced regulatory architectures from multi-omics data is a key AI application in plant systems biology [113]. Graph neural networks (GNNs) and related graph-based architectures have emerged as the most powerful tools for reconstructing hormone-regulated gene modules in forest species. They model nonlinear interactions and infer crosstalk edges, aligning directly with the complexity of ABA, JA, ethylene, and auxin signaling under drought, heat, and salinity stress [114]. This is exemplified by GNN-based miRNA–stress association prediction achieving AUPR 98.24% and AUC 97.43% across multiple woody species, identifying miR168, miR393, and miR396 as conserved regulators of ABA- and JA-linked stress pathways [96]. These findings validate the suitability of graph architectures for capturing hormone-responsive regulatory hierarchies in species with large, repetitive genomes. In other studies, Chang et al. [96] demonstrated this potential by integrating multi-source features into a GNN framework to predict abiotic stress-responsive miRNAs, providing a direct route for identifying post-transcriptional regulators of hormone signaling.

Additionally, node classification with GNNs enables prioritization of key regulatory hubs, such as SnRK2 kinases in ABA signaling, MYC2 in JA responses, and ARR-B-type regulators in CK-mediated drought tolerance, that are difficult to identify using expression data alone [115]. Edge prediction further uncovers previously uncharacterized protein–protein interactions, metabolite–gene associations, and cross-layer regulatory relationships. This includes mapping the ABA perception cascade from PYR/PYL receptor activation to PP2C inactivation, SnRK2 phosphorylation, and downstream transcriptional activation of stress-responsive genes [116]. Liu et al. [117] advanced this field by developing a multi-omics framework that integrates transcriptomics, proteomics, epigenetics, and network analysis to reconstruct cell type-specific drought regulation. Their incorporation of AlphaFold 3 structural predictions into models of CIPK–CBL complexes, key calcium-signaling components downstream of ABA perception, demonstrates how structural AI, protein-interaction modeling, and stress phenotyping can be unified within a single analytical pipeline. This cell-resolved approach provides a template for applying GNN-based hormone systems biology to forest tissues such as the vascular cambium, root apical meristems, and stomatal complexes under abiotic stress [117].

Co-expression network approaches (WGCNA) remain widely used in conifers, but AI-enhanced variants now provide deeper mechanistic resolution. In *Pinus tabuliformis*, network analysis identified ten ABA-integrating hub genes and 84 transcription factors responsive to drought [94], while Populus meta-analyses using Random Forest and SVM models highlighted MYB and MAPK nodes enriched for ABA- and SA-responsive cis-elements [13]. These studies demonstrate that machine learning-guided network inference can extract hormone-specific regulatory modules even from noisy or incomplete transcriptomes typical of forest species.

### 4.4. Protein Language Models: Decoding Stress-Responsive Hormone Proteomes

Proteomics under abiotic stress has been limited by incomplete annotation, high sequence divergence in non-model species, and difficulty predicting the functions of unannotated stress-responsive proteins [69]. Protein language models (PLMs), large-scale deep learning models trained on millions of protein sequences, address these limitations by learning statistical patterns that encode functional and structural properties, enabling inference of protein function without experimental annotation [118]. Models such as ESM-2, ProtBERT, and AlphaFold embeddings predict protein structure, hormone-binding motifs, and interactions for sequences without close homologs, greatly expanding the interpretability of proteomes in species lacking reference annotations [118].

In forest hormone biology, PLMs have enabled several critical advances. For ABA signaling, PLMs have predicted the structural domains of PYR/PYL receptors, PP2C phosphatases, and SnRK2 kinases in non-model species, enabling functional inference of stress-perception components across the phylogenetic breadth of plant diversity [119]. For JA and salicylic acid signaling, PLMs have identified COI1-JAZ interaction interfaces and NPR1 oligomerization domains under stress, supporting mechanistic analysis of hormone receptor complexes even in species with incomplete proteome annotations [120]. Phosphorylation site prediction by PLMs is particularly important for interpreting rapid hormone-mediated signaling events, as phosphorylation of ABA-responsive TFs, components of the MAPK cascade, and CDPKs underpins stress responses that occur within minutes of ABA perception [121]. The integration of PLM-derived embeddings with conventional proteomics pipelines, for instance, using ESM-2 embeddings as input features for stress-phenotype prediction models, represents an emerging strategy that bridges sequence-level biology with omics-scale functional analysis, reducing dependence on species-specific experimental annotations [47]. Examples of PLM techniques in forest hormone biology are summarized in Table 5.

### 4.5. Single-Cell and Spatial Multi-Omics: Resolving Hormone Gradients Under Stress

The application of single-cell RNA sequencing (scRNA-seq) and spatial transcriptomics to resolve cellular heterogeneity in hormone responses has transformed plant stress biology. Individual plant tissues under abiotic stress have cells in various states, differentiating, stressed, and hormone signaling, whose transcriptional identities are invisible at the tissue level [122]. scRNA-seq has revealed that abiotic stress responses are strongly cell type-specific: guard cells exhibit rapid, ABA-dependent stomatal closure, while mesophyll cells simultaneously engage in photosynthetic downregulation; vascular cells maintain hormone transport, while outer epidermal cells activate osmoprotectant biosynthesis [105]. Spatial transcriptomics adds a further dimension by preserving the positional context of gene expression within tissue architecture. Liu et al. [105] showed that ABA from root phloem acts as a paracrine signal, promoting cell-wall remodeling and barrier formation in outer cortical tissues under stress. This spatially organized response was elucidated by combining scRNA-seq with spatial transcriptomics. Coupling spatial datasets with AI models, including GNNs for cell communication, CNNs for spatial expression, and UMAP/t-SNE with clustering to identify stress-responsive states, offers a multi-resolution approach to map hormone gradients at single-cell precision [122]. In forest biology, combining single-cell and spatial transcriptomics with AI-based network inference advances the study of hormone systems. It helps distinguish cell-autonomous from non-cell-autonomous hormonal signals and their spread in tissues under stress.

### 4.6. Integrating Multi-Stress Scenarios and Hormone Network Crosstalk

Plants in natural environments rarely encounter stresses in isolation; instead, drought, heat, salinity, and cold occur concurrently or in rapid succession. These combined stresses produce emergent physiological states and regulatory interactions that cannot be inferred from single-stress datasets [123]. AI-augmented multi-omics offers a scalable way to resolve complexities by learning nonlinear interactions, integrating incomplete omics layers, and identifying regulatory nodes for hormone-mediated stress responses in forest species.

Multi-omics studies in conifers illustrate the value of such integrative approaches. In *Pinus tabuliformis*, transcriptome network analysis revealed hub genes that bridge ABA-dependent and ABA-independent pathways, with PtNCED3 strongly induced under prolonged drought, highlighting a central role for ABA biosynthesis in multi-stress acclimation [94]. Complementary metabolomics in *Pinus taeda* seedlings exposed to extended drought showed coordinated accumulation of ABA and salicylic acid in needles and GA_4_ in roots, defining tissue-specific hormonal adjustments that underpin conifer drought resilience [84]. Similarly, integrated transcriptome–metabolome analyses in Juglans regia demonstrated that methyl jasmonate activates antioxidant and phenylpropanoid pathways to enhance salt tolerance, underscoring the role of JA-mediated crosstalk in multi-stress defense [95].

Provenance-level variation further illustrates how multi-omics resolves divergent stress strategies in forest trees. Combined transcriptomic and metabolomic profiling of *P. tabuliformis* provenances identified distinct gene–metabolite correlation networks associated with jasmonic acid, proline, and flavonoid metabolism during drought and rehydration, revealing population-specific regulatory architectures [98]. Cross-species multi-omics comparisons extend this insight: integrated analyses in rice, maize, and tomato uncovered conserved metabolic signatures—such as phenolamide accumulation and flavonoid remodeling, regulated by JA–ABA crosstalk [124]. AI-driven integration of such datasets enables the transfer of conserved regulatory modules to non-model forest species, providing a predictive basis for stress-tolerance breeding and genomic selection.

Emerging frameworks now incorporate epigenetic stress memory, integrating ATAC-seq with transcriptomic and metabolomic profiles to reveal how prior stress exposure primes hormone-responsive regulatory circuits [122]. AI models trained on these multilayer datasets can infer chromatin-level determinants of durable stress tolerance, a feature particularly relevant for long-lived forest trees. In Populus, integration of transcriptomics with association genetics linked drought-responsive co-expression modules, enriched in ABA and auxin signaling, to underlying genomic variants, demonstrating how multi-omics can resolve the genetic architecture of hormone-regulated stress adaptation [90].

Multi-modal fusion models, capable of integrating any combination of omics and phenomics, are therefore essential for resolving hormone-regulated pathways across tissues and developmental stages. For example, integrated transcriptome–metabolome analyses in *Juglans regia* revealed MeJA-driven activation of phenylalanine and antioxidant pathways under salt stress [95], while *Pinus tabuliformis* drought–rehydration studies showed coordinated ABA (PYR/PYL-PP2C-SnRK2) and JA (AOC/AOS) pathway activation across organs [98], illustrating how fusion models uncover hormone-specific metabolic rewiring and organ-level divergence in stress strategies.

Across forest systems, AI-augmented multi-omics provides a mechanistically grounded, data-rich framework for decoding hormone-regulated stress biology. GNNs reconstruct regulatory hierarchies; VAEs and transformers capture latent and dynamic hormone states; fusion and causal models integrate cross-layer signals; and ML-based predictors link hormone pathways to adaptive phenotypes. Together, these approaches overcome long-standing barriers posed by large genomes, environmental heterogeneity, and complex stress–hormone interactions in woody species, positioning AI-driven multi-omics as a central engine for next-generation forest resilience research.

## 5. Conclusions and Future Perspectives

Multi-omics technologies have enhanced the understanding of forest hormone systems by enabling detailed profiling of omics layers. However, traditional methods struggle to resolve spatial–temporal hormone dynamics, model nonlinear interactions, and predict outcomes for forest species. AI offers transformative solutions through machine learning, deep learning, graph neural networks, and protein language models. These tools help integrate datasets, reconstruct hormone signaling, predict phenotypes under environmental stress, discover regulatory modules, infer hormone gradients, and model dynamic responses—outperforming conventional methods. The integration of AI with multi-omics data marks a crucial step in forest biology, providing insights into wood formation, cambial activity, dormancy, branching, and stress resilience. These advances are vital for climate-smart forestry, precision breeding, and sustainable management.

Integrating AI with multi-omics stands a potential to transform hormone systems biology in forest trees. Future efforts should focus on developing spatial multi-omics approaches that employ CNNs to learn spatial patterns from imaging and transcriptomics, thereby decoding hormone gradients and tissue polarity and supporting the reconstruction of auxin maxima in the cambium or of ABA hotspots during drought. GNNs may model tissues as cellular graphs, thereby reconstructing spatial regulatory networks that are crucial for understanding hormone signaling during wood formation or root development. Other multi-modal models might integrate omics data to infer hormone fluxes, transporter activity, and receptor distribution, thereby revealing hormone interactions, such as auxin-cytokinin or ABA, in stress responses. In addition, foundation models could transform forest hormone biology by enabling gene-function prediction in poorly annotated genomes, inferring regulatory modules from transcriptomic and epigenomic patterns, and identifying key nodes such as ARFs, DELLAs, and ARRs, even with limited data.

Protein language models could detect novel domains and hormone-binding pockets, advancing receptor and transporter discovery. As datasets grow, research in wood formation, stress resilience, and climate adaptation could become tools for predicting forest responses to environmental change, aiding breeding, restoration, and ecosystem management. Other tools, such as digital twins, may enhance forest systems biology by creating live replicas of hormone networks in trees; they integrate omics data with environmental factors, allowing managers to forecast growth, predict stress responses, and optimize practices like thinning and fertilization. In breeding, they may simulate plant responses to drought, heat, or nutrient stress, thereby accelerating trait discovery. They may model hormone responses to pests for early detection and predict changes in cambial activity to guide wood-quality decisions on density, fiber, and carbon use. Furthermore, ML models such as gradient boosting, neural networks, and ensembles can detect nonlinear interactions among loci, pathways, and the environment. In the future, these may accelerate the identification of superior genotypes for deployment or crossing, particularly when trials are costly and time-consuming.

Finally, integrating AI with multi-omics in forest hormone biology may increase collaboration among biologists, foresters, climate scientists, ethicists, and policymakers, aligning AI insights with ecological stewardship, community needs, and sustainability, for instance, in issues of respecting data sovereignty in indigenous forest regions or considering ecological risks.

## Figures and Tables

**Table 1:** Multi-omics datasets resolution on hormone-regulated processes in forest trees.

Omics	Application	Key Techniques	Hormone-Related Insights
Genomics	Gene content, regulatory elements, structural variation	WGS, long-read sequencing, pangenomes, GWAS, eQTL	Hormone-related gene families (PINs, ARFs, PYR/PYL), regulatory motifs, and adaptive variants
Transcriptomics	Gene expression dynamics	RNA-seq, Iso-Seq, time series, spatial transcriptomics, scRNA-seq	Hormone-responsive modules; tissue-specific gradients; hormone crosstalk networks
Proteomics/Phosphoproteomics	Protein abundance, PTMs, signaling complexes	LC-MS/MS, iTRAQ/TMT, SRM/PRM	Phosphorylation switches in ABA, BR, auxin pathways; receptor complexes
Metabolomics	Hormone levels, intermediates, metabolic shifts	GC-MS, LC-MS, NMR; targeted/untargeted	Quantifies hormone pools; maps gradients in cambium, buds; identifies crosstalk metabolites
Epigenomics	Chromatin state, heritable regulation	WGBS, RRBS, ChIP-seq, CUT&RUN, CUT&TAG	Links methylation/histone marks to dormancy, stress memory, and hormone-responsive genes

**Table 3:** Comparative analysis of traditional and AI-augmented multi-omics integration in plant hormone-mediated abiotic stress biology.

Feature	Traditional Multi-Omics Integration	AI-Augmented Multi-Omics Integration
Data handling	Manual alignment of omics layers; strong for targeted, pairwise comparisons	Automated fusion of heterogeneous datasets; enables high-dimensional, multilayer integration
Hormone crosstalk inference	Correlation- and rule-based networks; static and highly interpretable	Probabilistic and dynamic network inference (Bayesian models, GNNs) capturing nonlinear interactions
Abiotic stress modeling	Descriptive and enrichment-based analyses; limited predictive scope	Predictive modeling of single and combined stresses using supervised ML/DL
Stress–growth trade-off	Qualitative interpretation of resource allocation patterns	Quantitative simulation of hormonal trade-offs and adaptive strategies
Temporal dynamics	Time-series analyzed independently; limited resolution of lagged effects	RNNs, LSTMs, and dynamic Bayesian models capture phased hormone responses
Scalability	Effective for moderate datasets; manual curation ensures biological grounding	Scales to large, multi-tissue, multi-genotype datasets; transfer learning supports data-sparse systems
Interpretability	High transparency; directly linked to known pathways	Requires XAI tools (e.g., SHAP, attention weights) to interpret latent features
Single-cell and spatial resolution	Bulk omics; limited ability to resolve cell-type heterogeneity	Integrates scRNA-seq and spatial data to map hormone gradients at cellular resolution
Validation strategy	Experimental validation via hormone assays, qRT-PCR, phenotyping	AI-derived confidence scores and feature rankings streamline candidate prioritization
Forest system relevance	Establishes foundational hormone-stress maps in perennial species	Supports systems-level prediction and breeding for climate resilience

**Table 4:** AI approaches for multi-omics integration in plant hormone network signaling.

AI Method/Model	Multi-Omics Layers	Hormone-Signaling	Strengths	Limitations/Gaps
Graph Neural Networks (GNNs)	RNA-seq, metabolomics, epigenomics	Deducing hormone-regulated gene modules; reconstructing signaling cascades; predicting crosstalk edges	Captures network topology; models non-linear interactions; ideal for pathway reconstruction	Requires high-quality interaction graphs; limited interpretability
Deep Autoencoders/Variational Autoencoders (VAEs)	Transcriptomics, proteomics, interactomes, phospho-omics	Latent-space discovery of hormone response states; clustering hormone-specific signatures	Learns compressed representations; handles noisy, high-dimensional data	Latent features can be biologically opaque
Transformer Models	Time-series transcriptomics, chromatin accessibility, proteomics	Integrating hormone-responsive omics with phenotypes; predicting hormone-driven traits	Excellent for sequential and contextual data; captures delayed hormone effects	Data-hungry; computationally expensive
Multi-Modal Fusion Models	Any combination of omics + phenomics	Causal inference of hormone-regulated pathways; identifying upstream regulators	Flexible architecture; supports heterogeneous data	Fusion strategy selection remains non-standard
Bayesian Networks/Causal Models	Transcriptomics, metabolomics	Feature importance for hormone-responsive genes/metabolites; biomarker discovery	Explicit causal structure; interpretable	Sensitive to data sparsity; computationally intensive for large networks
Random Forests/Gradient Boosting	Targeted multi-omics panels	Identifying hormone-responsive cell states; mapping spatial hormone gradients	Robust to noise; interpretable feature rankings	Limited ability to model complex network dynamics
Clustering + Manifold Learning (UMAP, t-SNE)	Single-cell multi-omics	Identifying hormone-responsive cell states; mapping spatial hormone gradients	Excellent for exploratory analysis; reveals hidden structure	Not predictive; sensitive to parameter choices
Reinforcement Learning/Dynamic Models	Time-series omics + environmental data	Predicting hormone network adaptation under stress; optimizing hormone-driven phenotypes	Models dynamic decision-like processes; useful for stress-response modeling	Rarely applied; requires simulated environments

## Data Availability

No new data were created or analyzed in this study. Data sharing is not applicable.

## Abbreviations

The following abbreviations are used in this manuscript:

PLMs	Protein Language Models
GNNs	Graph Neural Networks
VAEs	Variational Autoencoders
RNNs	Recurrent Neural Networks
JA	Jasmonic Acid
SA	Salicylic Acid
GA	Gibberellin
ABA	Abscisic Acid
GIF	GRF-interacting factor
ACC	1-aminocyclopropane-1-carboxylic acid
IAA	Auxin
SWI/SNF	SWItch/Sucrose Non-Fermentable
LSTM	Long Short-Term Memory
SAR	Systemic Acquired Resistance
SVMs	Support Vector Machines
AI	Artificial Intelligence
RF	Random Forest
ML	Machine Learning
XAI	Explainable Artificial Intelligence
miRNAs	microRNAs
PIN	PIN-FORMED
ARFs	AUXIN RESPONSE FACTORS
ChIP-seq	Chromatin Immunoprecipitation Sequencing
CUT&RUN	Cleavage Under Targets and Release Using Nuclease
CUT&TAG	Cleavage Under Targets and Tagmentation
